# Automatic Guided Waves Data Transmission System Using an Oil Industry Multiwire Cable

**DOI:** 10.3390/s20030868

**Published:** 2020-02-06

**Authors:** Gianpiero Trane, Rito Mijarez, Jesús Arturo Pérez-Díaz

**Affiliations:** 1 Tecnologico de Monterrey, Escuela de Ingeniería y Ciencias, Colonia Real del Puente C. P. Xochitepec 62790, Morelos, Mexico; a01125467@itesm.mx (G.T.); jesus.arturo.perez@tec.mx (J.A.P.-D.); 2 Instituto Nacional de Electricidad y Energías limpias, Gerencia de Control, Electrónica y Comunicaciones, Calle Reforma 113, Col. Palmira C.P. Cuernavaca 62490, Morelos, Mexico

**Keywords:** piezoceramic transducers, guided waves-based communication, pulse position modulation, digital signal processing, multiwire cables

## Abstract

Alternative wireless data communication systems are a necessity in industries that operate in harsh environments such as the oil and gas industry. Ultrasonic guided wave propagation through solid metallic structures, such as metal barriers, rods, and multiwire cables, have been proposed for data transmission purposes. In this context, multiwire cables have been explored as a communication media for the transmission of encoded ultrasonic guided waves. This work presents the proprietary hardware design and implementation of an automatic data transmission system based on the propagation of ultrasonic guided waves using as communication channels a high-temperature and corrosion-resistant oil industry multiwire cable. A dedicated communication protocol has been implemented at physical and data link layers, which involved pulse position modulation (PPM), digital signal processing (DSP), and an integrity validation byte. The data transmission system was composed of an ultrasonic guided waves PPM encoded data transmitter, a 1K22 MP-35N multiwire cable, a hardware preamplifier, a data acquisition module, a real-time (RT) DSP LabVIEW (National Instruments, Austin, TX) based demodulator, and a human-machine interface (HMI) running on a personal computer. To evaluate the communication system, the transmitter generated 60 kHz PPM energy packets containing three different bytes and their corresponding integrity validation bytes. Experimental tests were conducted in the laboratory using 1 and 10 m length cables. Although a dispersive solid elastic media was used as a communication channel, results showed that digital data transmission rates, up to 470 bps, were effectively validated.

## 1. Introduction

Harsh environments characterize cased hole production engineering structures in the hydrocarbons sector, for example, downhole oil reservoirs either onshore or offshore could be immersed underwater or in corrosive liquids and high-temperature high-pressure boreholes’ surroundings [1]. Downhole data acquisition from a producing well is intended primarily to measure the performance of a well in production, better understand the dynamic behavior, plan remedial work if it is required, and optimize the long-term production. The production measurements are flow, density, temperature, and pressure. Hence, no hydrocarbon can be produced without the intervention of these measurements [2,3]. The acquisition of these bottom hole geophysical parameters using digital data transmissions under these aggressive conditions has become difficult, and the use of cables dedicated exclusively to communication media and conventional radio wave wireless communications has proven to be impractical [4,5]. The instrument that is descended into a wellbore to measure geophysical parameters must be able to endure the extreme hole conditions that could be encountered. The instrument is connected to a surface station, generally controlled by a computer, via a cable. Data acquisition should be performed with minimum disturbance to the normal operating production procedures. Detailed records, also termed well log, are surface read-out measurements that are achieved either in real time using electrical signals via power line communications and a multiwire cable as communication channel, or the data can be stored downhole in a bottom hole instrument operated by batteries and reviewed when it is retrieved later. In the former approach, downhole environmental factors, mainly high temperature, increase the electrical resistance in the cables and modify the behavior of the passive electrical components, for example, imposing important deviations in central frequencies of front-end filters, thereby compromising the propagation of electrical signals for data transmissions [6,7,8]. The advantages of the latter approach include lower operating costs and a fixed operations schedule. The main shortcoming is the possibility of important operating losses if the bottom hole recording instrument malfunctions. However, real-time downhole measurements that employ multiwire cables are recognized, in the petroleum industry, as more important for making prompt decisions [3].

Guided waves have been a relevant tool for non-destructive testing (NDT) and structural health monitoring (SHM) technologies due to their wide screening of the acoustic field and their ability to propagate long distances in large structures [9,10,11]. SHM of multiwire cables using ultrasonic guided waves has been investigated numerically and experimentally with a particular focus on damage detection [12,13,14,15,16,17,18,19]. Moreover, the reuse of metallic industry infrastructure has been proposed by several research groups as a communication medium for digital transmission systems using field-proven guided waves that are excited and received either by piezoelectric or electromagnetic transducers. The methods employed for modulation include frequency-shift keying (FSK), amplitude-shift keying (ASK), chirp on-off keying (Chirp-OOK), pulse position modulation (PPM), orthogonal frequency-division multiplexing (OFDM) and code-division multiple access (CDMA), among others. Some researchers have used metal pipes [20,21,22,23,24,25,26,27], thick metal barriers [28,29,30,31,32,33,34,35,36], and plates [37,38,39,40,41,42] as communication channel. 

Recently, the use of cables as a communication medium was proposed by Mateo et al. [43,44]. They used a single copper core PVC insulated electrical cable type H07V-U and two piezo ceramic discs, one as a transmitter and the other one as a receiver. The transmitter was excited via a train of chirp signals in the range of 1 to 12 MHz and a signal averaging process was performed in the received signals. They achieved short-range communication of 0.25 m using a PVC insulated cable and 0.50 m by removing its dielectric PVC covers. Copper stranded cable 12 rating American wire gauge (12 AWG) has been used as a communication medium in a previous study [45,46]. In this approach, a digital data transmission system was developed that performed pulse position modulation (PPM) in the range of 60 kHz, to transmit eight bits of encoded information over a 12 AWG multiwire cable that was 1 m and 4 m in length. The aim was to achieve digital transmission of guided waves on mono-conductor multiwire cables commonly used in producing wells. The researchers also carried out preliminarily work in 1 m corrosion-resistant four conductors 4H18 multiwire cable, in which data rates in the order of 500 bits per second were accomplished by employing tone pulses of 60 kHz and PPM [47]. 

In this study, we proposed a downhole instrument that is able to periodically transmit ultrasonic guided waves signals via a multiwire cable indicating that the instrument is operating correctly and sending complementary information related to the recorded bottom hole parameters. Hence, high-pressure and high-temperature corrosion-resistant mono-conductor multiwire cables 1K22 MP-35N were employed. The periodicity of transmitted data, in this application domain, was either once every hour at the beginning of its operation or once per day when it reached a stable state at the bottom hole, depending on the operator requirements. Thereby, there was no need for stringent high data rates, and several transmitted bytes of information could suffice. A progressive approach was taken for the use of PPM and guided waves on this intricate mono-conductor multiwire cable. First, we achieved results using a low-cost electrical copper stranded cable 12 rating American wire gauge (12 AWG); secondly, we moved the investigation to a corrosion-resistant four-conductor cable 4H-18 and guided waves; and finally, we used the intended mono-conductor 1k22 MP-35N cable. The system consisted of an ultrasonic guided waves PPM encoded data transmitter, a hardware preamplifier, a data acquisition module, a real-time (RT) DSP LabVIEW based demodulator, purchased from National Instrument (NI) Mexico, and a human-machine interface (HMI) running on a personal computer. A dedicated communication protocol was implemented at physical and data link layers, which involved PPM, digital signal processing, and an integrity validation byte. To evaluate the communication system, the transmitter generated 60 kHz PPM energy packets containing three different bytes and their corresponding integrity validation bytes. Experimental tests were conducted in the laboratory using 1 and 10 m length cables. Automatic digital data transmission rates up to 470 bps were successfully achieved. In this paper, the panorama of research and detailed significant characteristics of the novel automatic data transmission guided waves system is described. The proprietary hardware design of the original battery-operated stand-alone electronics instrumentation for the guided waves transmitter is presented, including its schematic diagram; and on the receiver side, the performed signal processing algorithms are detailed.

## 2. Guided Wave Propagation in Multiwire Cables

**Figure 1:**
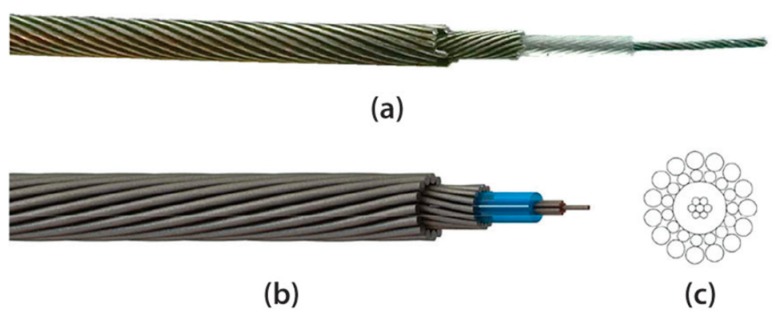
(**a**) Actual 1K22 MP-35N corrosion-resistant cable; (**b**) 1K22 MP-35N CAD model; and (**c**) 1K22 MP-35N construction.

Multiwire cables are more complicated mechanical structures than individual solid cylindrical rods. The complex interaction between multiple wires due to the geometry of its cross-section is its most important feature. 1K22 MP-35N cables are multiwire structures made of an inner copper construction, a plastic insulation layer, and a steel armor that can withstand high-temperature, high-pressure, and corrosive environments. Figure 1a,b shows an actual 1K22 MP-35N cable and CAD model, respectively, and Figure 1c illustrates the composition of the wires. The inner construction is comprised of seven individual nickel-coated copper 18 AWG wires of 0.404 mm diameter. Voids in the copper strands are filled with a water-blocking agent to reduce water and gas migration. The copper construction is covered in a perfluoroalkoxy (PFA) plastic-type isolation layer that is 0.713 mm in thickness. The armor is made up of two types. The former is an inner armor of 15 wires that are 0.617 mm in diameter, and the latter is an outer armor of 15 wires that are 0.909 mm in diameter. Both armors are nickel-cobalt corrosion-resistant stainless-steel wires, which are coated with an anti-corrosion compound for protection during shipping and storing. A special pressure seal agent, SuperSeal (Canusa-CPS, Toronto, ON, Canada), is applied between the armor layers [48].

To the best of our knowledge, an analytical solution that describes the wave propagation in these complex multiwire cables does not exist. A practical approach to understand guided wave propagation in multiwire cables is based on the study of wave propagation behavior of an individual wire using the analytical Pochhammer–Chree frequency equation which has been discussed in detail by Graff [49] and more recently by Rose and Nagy [50]. The solutions of the elastic equation of motion yield an infinite number of guided waves modes, which are categorized by their particle displacement, and can well propagate through the structure. These modes in a cylindrical waveguide correspond to the longitudinal waves L(0,n) with radial and axial vibration displacement, torsional waves T(0,n) with circumferential vibration displacement, and flexural waves F(M,n) with radial, axial, and circumferential vibration displacement, where M refers to the circumferential order and n represents the nth root of the characteristic equation. All possible propagating wave modes in a cable at different frequencies can be predicted by guided waves dispersion curves, which can be obtained by solving the Pochhammer–Chree frequency equation, via the commercial software Disperse [50].

**Figure 2:**
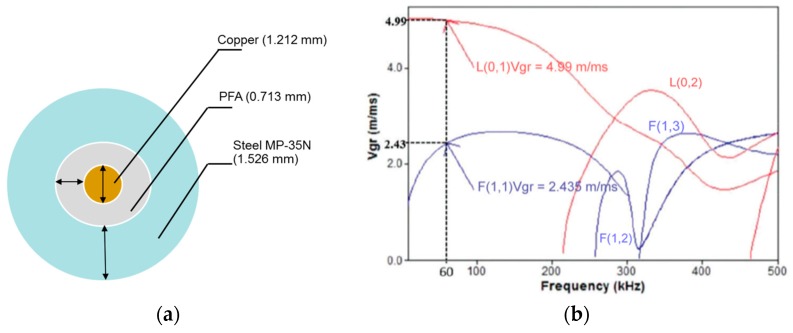
(**a**) Cable cross-section considerations to solve the Pochhamer–Chree frequency equation; (**b**) dispersion curves of a 1.212 mm diameter copper rod surrounded by a perfluoroalkoxy (PFA) of 0.713 mm thickness and steel armor of 1.526 mm thickness.

The approach taken by researchers of previous studies consisted of modeling two different multiwire cables, an AWG 12 cable and a 4H18 cable, as a concentric rod composed of different materials [45,47]. The model considered that the wavelengths of the propagating guided waves employed was significantly larger than the small gaps between the strands. Cheng et al. also modeled, in this manner, an aluminum conductor steel reinforced (ACSR) multiwire cable [18]. In this work, a multiwire 1K22 MP-35N cable was modeled as a rod made of copper that was 1.212 mm in diameter, surrounded by an isolation layer of PFA that was 0.713 mm in thickness, and coated with steel armor that was 1.526 mm in thickness, as depicted in Figure 2a. The group velocity dispersion curves for this concentric rod calculated by the software Disperse are plotted in Figure 2b.

### Frequency and Signal Selection for Guided Waves Excitation

The propagation of guided waves has been researched in three regimens, short (<< 1 m), medium (up to about 5 m), and long range (above 100 m). In long-range structures, frequencies below 100 kHz are generally required, as validated by Cawley et al. [51]. However, when working with frequencies lower than 100 kHz, the audio bandwidth that can lead to interference and a poor SNR. Studies have been carried involving the propagation of 20 and 40 kHz guided waves energy packages in tubes [21,23] and 60 kHz guided waves pulses in electrical 12 AWG cables [45,46], and preliminarily in 4H18 multiwire corrosion-resistant cables [47]. As shown in Figure 2b, for a concentric rod copper-PFA-steel, below 200 kHz, the assortment of the modes presents less difficult since there are only two possible excited modes, L(0,1) and F(1,1). Hence, the frequency selection depends on the transducer device and its frequency spectrum. In this work, piezo ceramic discs with normal beam loading and reception have been employed, thereby it is anticipated that longitudinal modes and flexural modes are excited depending on the pressure distributions exerted on the surface of the cable.

NDT and SHM long-range applications commonly use narrowband signals to provide suitable signal strength and prevent dispersion. Single-frequency sinusoidal waves of between five and ten cycles, modulated by a Hanning or a Gaussian window, are often employed [51,52]. The use of square pulses produces an increase in the energy of the transmitted signals enhancing the SNR, as has been validated by Pollakowski et al. [53]. Square pulses contain higher harmonics that can produce different speeds than the carrier frequency, which can lead to dispersion in an elastic media and multiple mode excitations. However, piezoelectric (PZT) transducers possess a frequency response similar to band-pass filters, with a bandwidth that depends on the transducer design. Hence, only the carrier frequency plus the frequency components that enter the transducer bandwidth can go through. In this application, the use of square wave pulses to excite PZT elements that possess a narrow bandwidth (3 Hz) yields an additional gain to the transmitted guided waves signal. The electronics required for the generation of square wave pulses presents two advantages that simplify its design. First, the square waves can be achieved readily using high-speed and highly integrated digital circuits such as microcontrollers, avoiding the employment of digital-to-analogue converters and low pass filters. Secondly, the power amplifiers that are normally used to increase the voltage signals applied to PZT elements, can be substituted for simple signal boosters, for example, H-bridge driver circuits. Considering these aspects and the experience of researchers in previous works on multiwire cables, square pulses of 60 kHz were used for powering piezo ceramic PZT-5H elements.

## 3. Pulse Position Modulation Communication

**Figure 3:**
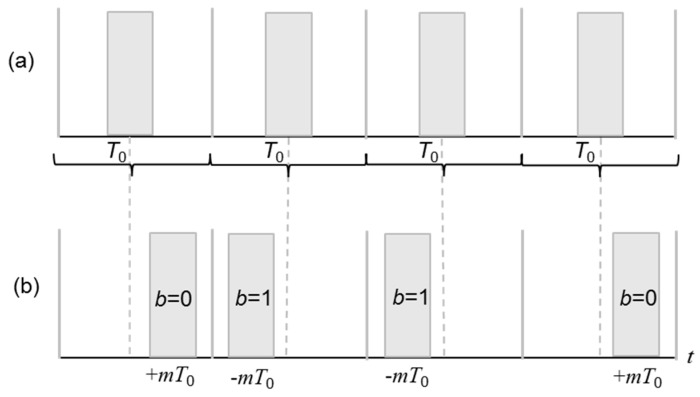
(**a**) Sequence of pulses without modulation and (**b**) sequence of pulse position modulation (PPM) pulses (0110).

PPM is an efficient modulation technique that has been used by several research groups to transmit digital information via guided waves [23,24,27,47]. In this application, PPM has been applied in the decoding and encoding process to attain data transmission using guided waves through multiwire structures. PPM is an attractive modulation due to its reduced sensitivity to multipath propagation; its simplicity for power-efficient channels, theoretically, PPM systems are effective when the signals are power limited rather than band limited, as a result of the transmission of k bits for the same average power [54]; and the instrumentation required for its implementation is simple and lightweight. PPM is a modulation scheme in which the message information is modulated in the time slot between pulses, *M*, in a sequence of signal pulses. In PPM, a symbol can be represented by

(1) $$k=log_{2}\left(M\right)$$

where *k* is the number of bits. A symbol contains a single pulse in the position indicated by the binary representation of the data codeword, thus, *k*/*M* bits are transmitted per time slot [55]. In general, the PPM generation of signals, *S_m_*(*t*), is given by [56]:

(2) $$S_{m}\left(t\right)=\varphi \left(t-mT_{0}\right);\;m=\frac{{\left(-1\right)}^{b}}{2}$$

where *φ*(*t*) is a unit energy pulse that is similar to those found in the sampling theorem, *T*_0_ is the time slot for each digital bit representation, and m stands for the temporal displacement of an acoustic pulse quantified for half of the time slot *T*_0_. The coefficient b represents the digital value of each bit transmitted within its time slot *T*_0_. The direction of the temporal displacement m, from −*T*_0_ to *T*_0_, is given by the digital value of the transmitted bit. For displacements with an increment of time, +*m*, the modulation corresponds to the logical level zero (bit 0) and for displacements with a decrement of time, −*m*, the modulation represents the logical level one (bit 1). As an example, a sequence of pulses that are not modulated is depicted in Figure 3a, where each pulse is exactly in the center of a time slot. Figure 3b shows a sequence of pulses displaced ±m, according to the PPM in a 0110 sequence of bits.

## 4. Automatic Guided Waves Data Transmission System

**Figure 4:**
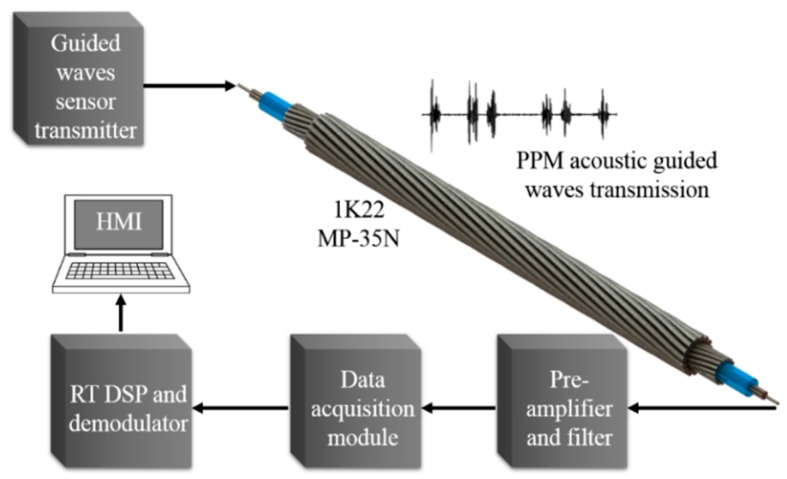
Automatic data transmission guided waves communication system block diagram.

The overall automatic guided waves data transmission system is illustrated in Figure 4. The system consists of a microcontroller-based piezo-ceramic transmitter as PPM generator, 1K22 MP-35N multiwire cable as communication channel, and an amplifier and a commercial data acquisition LabVIEW based modules connected to the HMI running in a computer as PPM receiver for automatic PPM demodulation.

### 4.1. PPM Signal Transmitter

**Figure 5:**
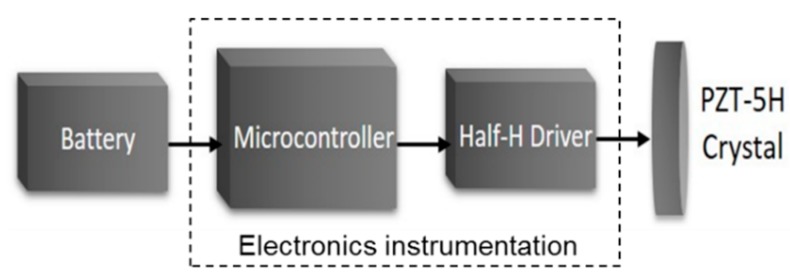
Block diagram of the PPM signal generator design used as a transmitter.

The PPM generator, working as guided waves transmitter, is a stand-alone battery-operated electronic module composed of a microcontroller, a half-H driver, and a piezo ceramic PZT-5H disc, as is shown in Figure 5.

**Figure 6:**
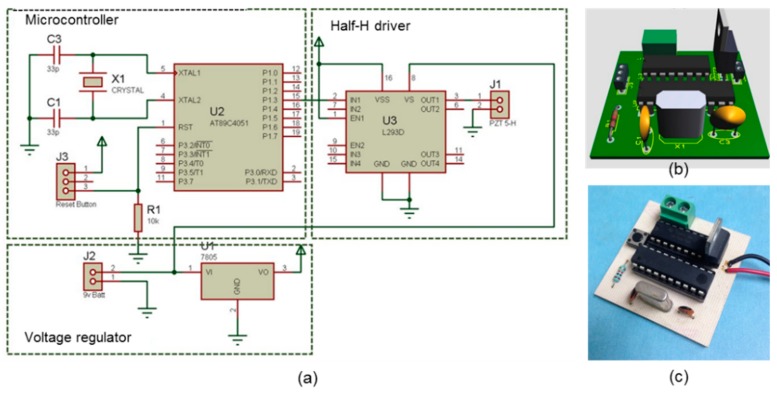
Electronics design and construction of the PPM generator. (**a**) Schematic diagram; (**b**) PCB design; and (**c**) actual PPM generator.

The electronics instrumentation is a simple design that possesses relatively little weight. The electronics circuit is made of three integrated circuits, i.e., a microcontroller, a half-H driver, and a voltage regulator. Figure 6 depicts its schematic diagram, printed circuit board (PCB) design, and the actual PPM generator.

**Figure 7:**
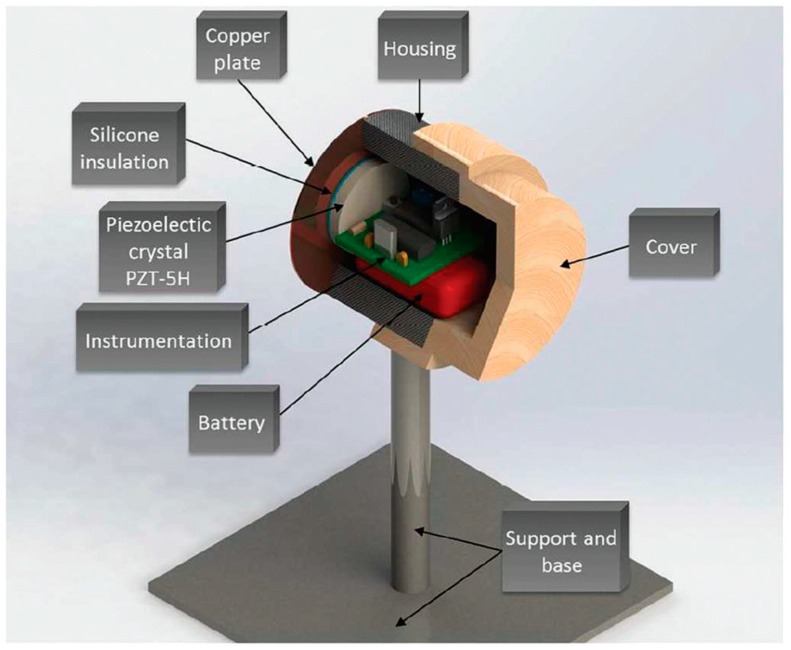
PPM generator components.

The components employed in the assembly of the PPM generator are a 9 V battery, the electronics instrumentation, and the PZT-5H crystal disc 38 mm in diameter and 5 mm in thickness, which were mounted in a cylindrical case made of a polymer called Nylamid (Nylene, Wayne, NJ, USA) with an inner diameter of 2 inches and an outer diameter of 3 inches. The case was fixed to a metallic support and base, as depicted in Figure 7.

The PZT-5H crystal connected to the electronic instrumentation was glued using silicone to an external copper plate 3 inches in diameter and 1 mm in thickness. One side of the copper plate was fixed to the case and the other was soldered to the copper cables of the 1K22MP-35N cable. Details of the PPM generator construction have been previously described by [57].

The program that generates the PPM encoded words was programmed into the internal flash memory of the microcontroller in the instrumentation module. By using a time slot of *T*_0_ = 1998 μs, according to Equation (2), the PPM digital construction for a logic zero and logic one is computed as:

(3) $$mT_{0}=\frac{{\left(-1\right)}^{0}}{2}\left(1998\right)=999\;\mu s\;mT_{0}=\frac{{\left(-1\right)}^{1}}{2}\left(1998\right)=-999\;\mu s$$

**Figure 8:**
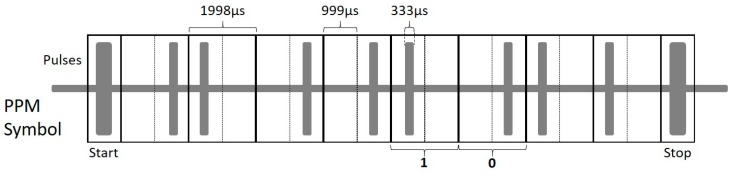
PPM symbol codification scheme for a sequence of 01001011 (4BH) data.

The construction of a logical one is defined by the position of the energy pulse to the first half of the time slot, *mT*_0_ = −999 μs. Correspondingly, the second half of the time slot *mT*_0_ = 999 μs determines the logical zero. The microcontroller continuously generates 10-bit frames of guided waves PPM encoded energy pulses. The frame is comprised of a start pulse, eight pulses of data, and a stop pulse. The start and stop bits that delimitate each byte are made of 40 square pulses of 60 kHz, i.e., 666 μs. After the start bit, there is a set of 8 bits made of 20 square pulses of 60 kHz, i.e., 333 μs width each, carrying the actual digital information. Figure 8 illustrates this PPM symbol scheme.

### 4.2. PPM Signal Receiver

**Figure 9:**
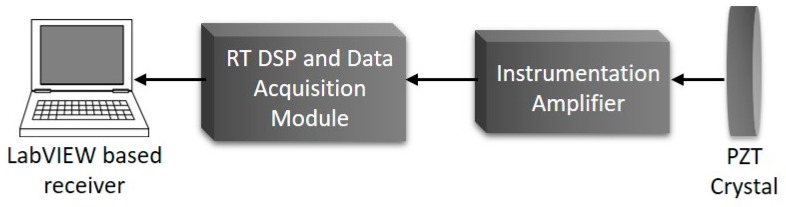
PPM guided waves receiver instrumentation block diagram.

The PPM guided wave receiver instrumentation package consists of a PZT-5H crystal, an instrumentation amplifier acting both as an amplifier and filter, a real-time LabVIEW based demodulator and data acquisition module, and an HMI used to set the system parameters and plot the results. Figure 9 depicts a block diagram of the PPM guided waves receiver instrumentation. A general description of the PPM receiver construction was recently carried out by [58].

One face of the PZT-5H crystal is connected to a low noise (8ηV/√HZ) instrumentation amplifier that also performs as a low pass filter with a gain of ten and a cut-off frequency of 500 kHz [59]. The instrumentation amplifier and the PZT-5H crystal are mounted in a cylindrical housing case such as the PPM transmitter. The other face of the PZT-5H disc is silicon glued to one side of a copper plate, and the other side is soldered to the other end of the central 1K22 MP-35N copper cables. The output of the instrumentation amplifier is connected to a commercial NI data acquisition module, which has a 16-bit analogue-to-digital converter with a ±10 V analogue input and a sample rate up to 1 MHz. This module is interfaced to a real-time (RT) NI CompactRIO (cRIO) controller, which is used to acquire the PPM guided waves signals. The cRIO controller possesses a processor and programmable FPGA. This controller is optimized for programming code with LabVIEW that can be deployed onto both the RT operating system and directly onto the FPGA. The PPM demodulator and the digital signal processing algorithms run over the cRIO FPGA, which also is used to transmit the results and information to a personal computer via Ethernet to form the IHM.

#### Digital Signal Processing, Signal Digitalization, and PPM Demodulation

**Figure 10:**
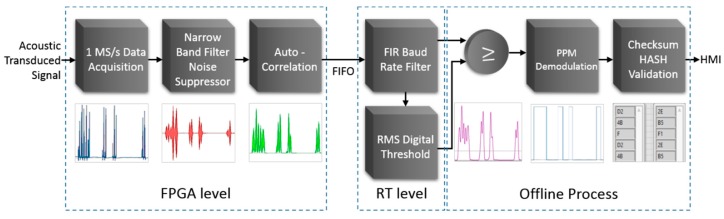
PPM digitalization and demodulation processes from data acquisition of the acoustic signals to byte validation.

The PPM digitalization and demodulation processes are performed sequentially in three segments as depicted in the block diagram of Figure 10. First, the FPGA of the cRIO controller executes in real time a 1 MS/s data acquisition of the guided waves signals and carries out digital signal processing (DSP) operations such as a narrow band-pass filter and an autocorrelation-based function for noise suppressing and signal improvement. The blue signal depicted in Figure 10 represents the acquired signal coming from the instrumentation amplifier. The red curve stands for the output of the narrow band-pass filter, and the green signal embodies the autocorrelation of the filtered signal. In the second segment, the following two RT DSP operations are performed: a low pass finite impulse response (FIR) filter tuned to the transmission baud rate and a root mean square (RMS) threshold computation. The third segment of the process is accomplished offline to display information in the IHM. At the start, a comparison between the baud rate of the filtered signal and the RMS threshold value is carried out, subsequently, a post-processing PPM demodulation and checksum HASH byte validation are implemented. The magenta curve represents the output of the baud rate tuned filter and contains a horizontal crossing line that stands for the RMS threshold value. The cyan curve shows the digital signal in which every pulse represents the duration of each acoustic pulse, and the time between them conveys the PPM information. Finally, two arrays display in hexadecimal the actual PPM received data and their validation HASH byte.

The narrow band noise suppressor filter implemented in the RT FPGA performs a band-pass infinite impulse response (IIR) Butterworth filter, whose cut-off frequencies are 48 and 62 kHz. The general difference equation that characterizes an IIR filter is given by [60]

(4) $$y_{i}=\frac{1}{a_{0}}\left(\overset{N_{b}-1}{\underset{j=0}{\sum }}b_{j}x_{i-j}-\overset{N_{a}-1}{\underset{k=1}{\sum }}a_{k}y_{i-k}\right)$$

where *b_j_* is the set of forward coefficients, *N_b_* is the number of forward coefficients, *a_k_* is the set of reverse coefficients, and *N_a_* is the number of reverse coefficients; and where *x_i_* is the current input, *x_i−j_* is the past inputs, and *y_i−k_* is the past outputs.

Signal enhancement is attained by using an autocorrelation-based function, which can be expressed by [61]

(5) $$y\left(l\right)=\sum_{n=i}^{\langle N-|k|-1\rangle }x\left(n\right)x\left(n-l\right)$$

where *i* = *l*, *k* = 0 for *l* ≥ 0, and *i* = 0, *k* = *l* for *l* < 0. Index *l* is a time shift or lag parameter. The disposition of this function presents two advantages: to stand out the guided waves pulses amplifying acoustic signal groups and to attenuate noise. The practical implementation of this operation is performed calculating the product of the received filtered signal *x*(*n*) multiplied by itself delayed with a defined temporal displacement *x*(*n* − *l*). The autocorrelation-based function multiplies positive slopes by themselves amplifying positive peaks. It also multiplies negative slopes by themselves that results in positive values amplified as positive peaks. As a result, the noise stays at a low amplitude level, while the acoustic pulses are amplified. This facilitates the identification of the acoustic pulses and provides well-defined amplified positive acoustic pulses ready to be filtered with a low-pass filter to unify them into a single pulse representing the width of a digital square pulse modulated in PPM.

The baud rate tuned filter implemented is the 129 taps FIR low-pass filter that is executed using the standard FIR convolution expression [61]

(6) $$y\left[n\right]=\overset{N-1}{\underset{k=0}{\sum }}h\left[k\right]x\left[n-k\right]$$

where *y*(*n*) and *x*(*n*) correspond to the output and input signals, respectively, and the filter impulse response is denoted by *h*(*k*). The coefficients of the filter are obtained using the frequency sampling method, whose algorithm is condensed by the expression

(7) $$h\left[n\right]=f_{w}\left[n\right]F^{-1}\left\{H\left[k\right]\right\}\{\begin{array}{l} H_{r}\left[k\right]=1,\;f_{l}<k<f_{h}\\ H_{r}\left[k\right]=0,\;elsewhere\\ \;H_{i}\left[k\right]=0 \end{array}$$

where *f_w_*[*n*] denotes a window function and *H*[*k*] characterizes the frequency response of the filter specified in the Fourier domain. The frequency response, for a linear phase filter, is determined by setting the real terms (*H_r_*[*k*]) to their intended values denoted by the low and high cut-off frequencies defined by the terms *f*_l_ and *f_h_* and specifying the imaginary terms *H_i_*[*k*] as zero. The real-time digital filter was implemented with a bandwidth of 80 Hz, whose cut off frequencies were 0 Hz and 80 Hz, respectively, i.e., it was designed to operate to the actual transmission baud rate.

After the baud rate FIR filter is executed, a RMS function is computed using 30 k samples for the digitalization process. The threshold value is characterized through the RMS value expressed by [62]

(8) VRMS=1N∑i=0N−1(xi )^22

where the values *x_i_* are the samples of the periodic signal and exact N samples are equally spaced along one complete signal period.

Signal digitalization is attained by carrying out a comparison point-to-point between the baud rate low-pass filtered signal and the RMS threshold values. The resultant digital pulses accommodate the transmitted PPM encoded information. The demodulation processes commence by identifying the start and the stop pulses and quantifying the time delay between the previously digitalized pulses. Each bit depends on the digital value of the previous bit and the delay time between them. Discounting start and stops pulses, the demodulator identifies frames of 8 bits. The decoded bytes are stored in a two-dimensional array of 8 Booleans values per row that contain the actual transmitted bytes and their corresponding validation HASH bytes. The validation process separates the actual data bytes from the validation HASH bytes and performs the HASH calculation and validation. Demodulated data bytes are converted to hexadecimal bytes and stored in a one-dimensional (1D) array. Decoded HASH bytes are compared with their correspondent data bytes to identify transmission errors.

## 5. Experimental Configuration and Automatic PPM Communication Results

**Figure 11:**
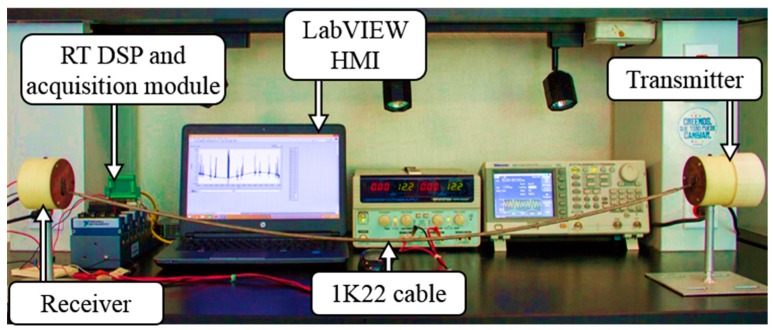
Experimental setup.

A series of experiments were conducted in the laboratory using two lengths of 1K22 MP-35N multi-conductor cables. The first set of experiments were aimed to identify the excited guided waves modes, L(0,1) and F(1,1), using a 1 m long cable. The second group of trials was conducted using 1 and 10 m long cables to automatically perform the transmission and reception of PPM symbols of 8-bit digital information. Each end of the 1K22 MP-35N inner copper multiwire cables, 18-AWG, was soldered to the 3 inch diameter copper plates mounted and fixed to the transmitter and the receiver instrumentation modules, respectively. The experimental setup for both experiments is illustrated in Figure 11.

### 5.1. Guided Wave Propagation Mode Identification

**Figure 12:**
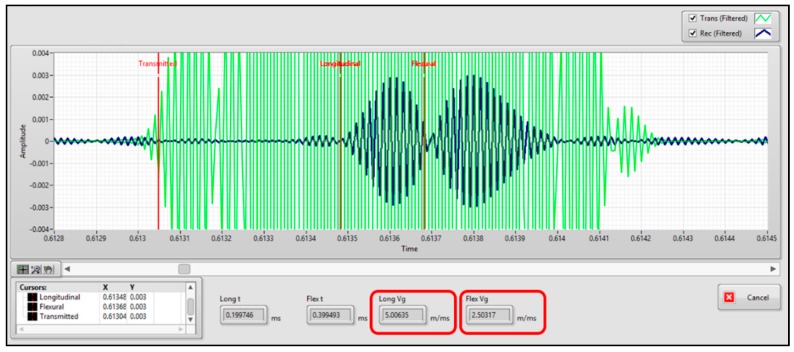
Guided wave propagation mode identification of a group of signals transmitted through 1 m of 1K22 MP-35N cable. The pale green curve shows the transmitted pulses and the dark green curve shows the excited guided waves modes.

In this experiment, the transmitter generated 20 square pulses of 60 kHz. A time domain analysis estimation of the time-of-arrival (TOA) of the received signal was implemented. The time difference between the transmitted and the received signal, over 1 m cable, gave an approximation of the propagation group velocity. Figure 12 illustrates both the transmitted square pulses (pale green curve) and the guided waves signals received signals (dark green curve). 

Two groups of signals with different group velocities were received. By using the LabVIEW HMI, it was observed that the first group of signals propagated at 5.00635 m/ms, and the second group propagated at 2.50317 m/ms. According to the dispersion curves in Figure 2b, two guided waves can be excited at 60 kHz, one at 4.999 m/ms, and the other at 2.435 m/ms. The received first group agrees to the longitudinal mode L(0,1), and the second group concurs to the flexural mode F(1,1); thereby it is possible to recognize the excited guided waves modes.

### 5.2. Automatic Guided Waves PPM Communication Results

**Figure 13:**
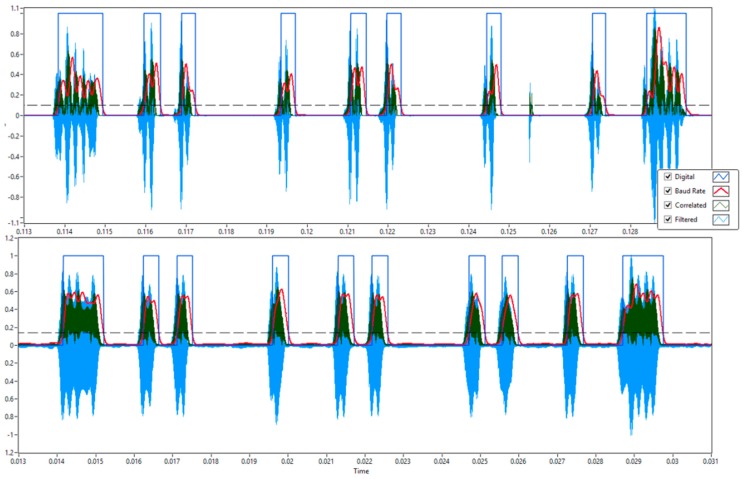
Digital signal processing (DSP) and signal digitalization of a symbol (4BH) identification over 1 m cable, upper trace, and 10 m cable, lower trace.

In the second group of experiments, two lengths of multiwire cables were employed, 1 and 10 m. The flash memory of the PPM instrumentation transmitter was programmed with three different symbols that were transmitted continuously once the instrumentation was powered. Digital signal processing and signal digitalization y PPM demodulation were performed automatically in the receiver instrumentation package. Figure 13 depicts the received DSP and signal digitalization process when the transmitter sends a symbol 4BH (01001011B).

In both plots, cyan curves are the received acoustic guided waves signal band-pass filtered; green curves, are the correlated signal; red curves, are the baud rate filtered signal; the horizontal dotted black line is the RMS value; and the blue square pulses are the actual digital data encapsulated in an 8-bits symbol, including start and stop pulses. The attenuation between the 1 and 10 m transmissions is about 32 dB; nevertheless, ultrasonic pulses are more uniform in the 10 m cable avoiding signal minimums under the threshold. In this case, the baud rate filter generates a smooth and thick positive peak (red curve) for each group of acoustic pulses. Although the energy loss is higher in the 10 m cable than in 1 m cable, the system provides sufficient SNR to successfully decode and demodulate the transmitted information.

**Figure 14:**
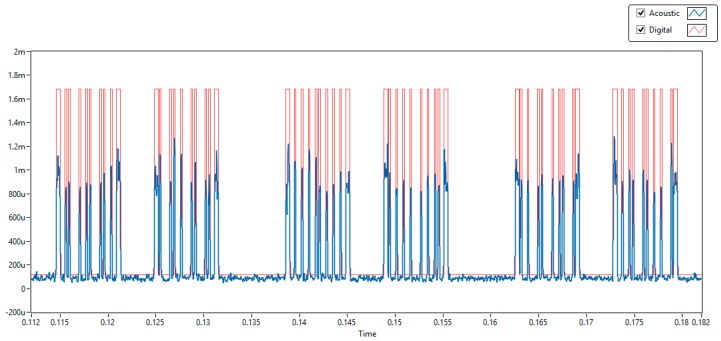
Identification of three different information bytes and their corresponding HASH bytes.

The visualization of the intermediate DSP processes was omitted in the final tests to accelerate its processing at the FPGA level. Figure 14 shows six of groups of signals; in blue curves, the DSP acoustic signals, and in red curves, the digitalized acoustic pulses. These groups of signals correspond to the transmission of the three information bytes and their associated HASH bytes delayed 10 ms. Each information byte and HASH byte are transmitted every 20 ms.

**Figure 15:**
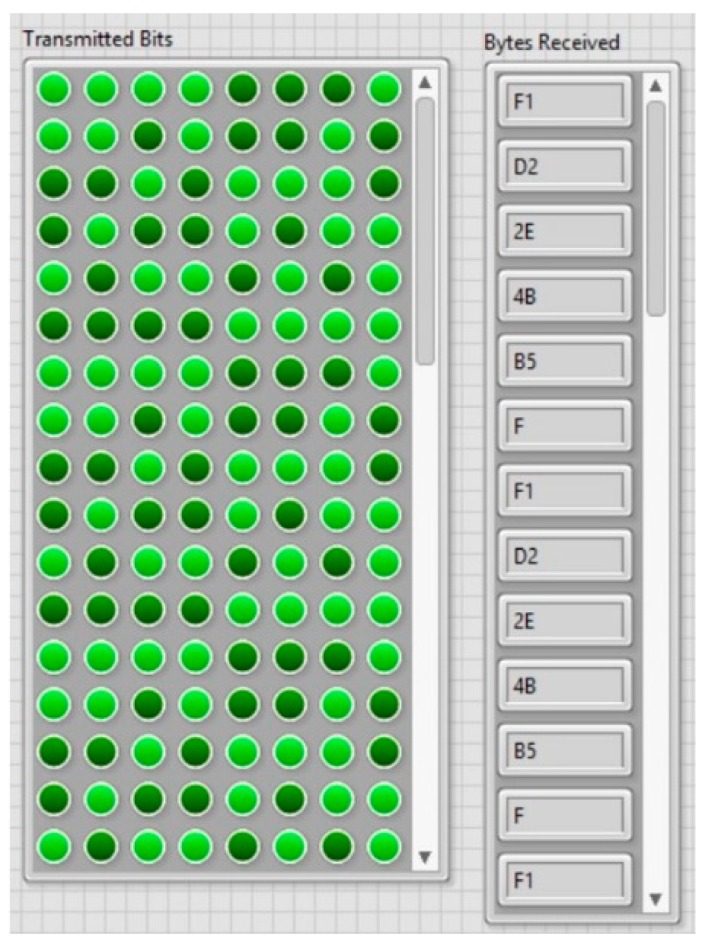
Two-dimensional (2D) array of 8 digital bits transmitted per row and its hexadecimal values of the received bytes.

The automatic PPM demodulation results were visualized using the IHM developed in LabVIEW. Figure 15 depicts a two-dimensional (2D) array containing 8 bits per row, where the LSB (least significant bit) is located on the right side of the row. Every row represents the byte information that is continuously transmitted. The hexadecimal values of received bytes per row are stored in an array and displayed in the IHM.

**Figure 16:**
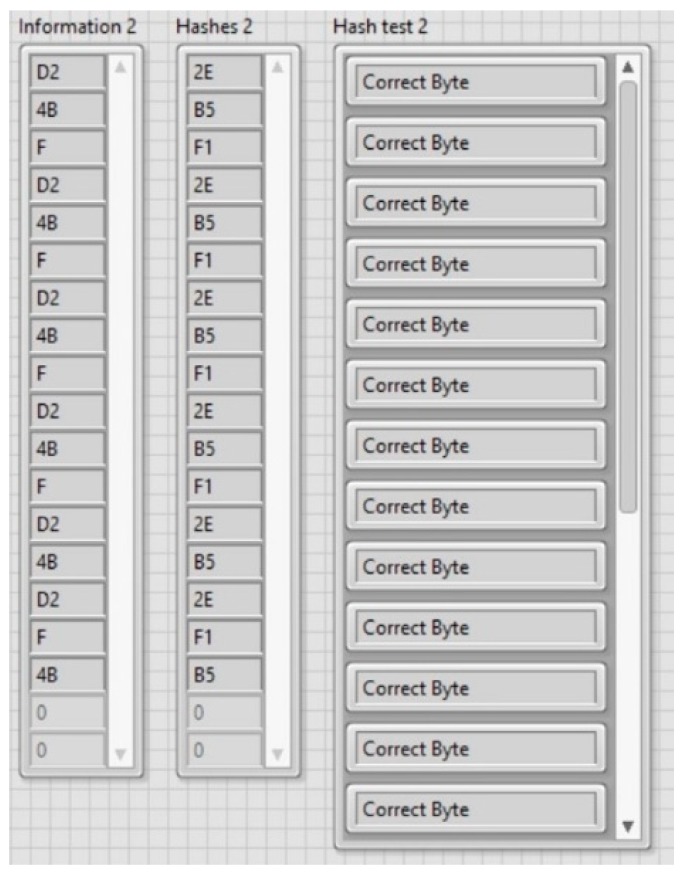
Received information bytes with its corresponding HASH bytes, and HASH validation.

Figure 16 shows the transmitted information byte and its HASH byte, in two separated arrays. The HASH test validation results are shown in a separate string array. Each element of the array contains the string “Correct Byte” or “Transmission Error” depending on the equivalency between each data transmitted byte and its HASH byte.

As depicted in Figure 15 and Figure 16, the automatic PPM demodulation process successfully identified, validated, and displayed the transmitted information in real time using 1 and 10 m 1K22 MP-35N multiwire cables as a communication channel. Data transmission rates, up to 470 bps, were experimentally obtained, which correspond to the selected acoustic propagation frequency of 60 kHz. The average byte error rate of the communication system was around 5% for 10 m transmission under laboratory conditions.

## 6. Discussion and Further Work

An automatic ultrasonic guided waves PPM data transmission system using high-temperature, high-pressure, and corrosion-resistant 1K22 MP-35N multiwire cables as a communication channel has been designed, implemented, and evaluated. The system uses cables commonly employed in the hydrocarbon industry for downhole data acquisition from producing wells, which are often immersed in harsh environments. The complicated mechanical structures of the 1K22 MP-35N multiwire cable was modeled as concentric rod made of copper, an isolation layer and steel. Dispersion curves of this concentric rod were calculated using the software Disperse. The identification of guided waves modes, L(0,1) and F(1,1), at 60 kHz and over a 1 m multiwire cable, were validated in the laboratory. 

A battery-powered stand-alone instrumentation PPM transmitter made of a microcontroller, a signal booster, and a piezo ceramic PZT-5H disc was implemented. Three different byte-information symbols with frames of 10 bits were programmed in the internal flash memory of the microcontroller and were transmitted continuously once the instrumentation was powered. The receiver instrumentation package consisted of a PZT-5H disc, an instrumentation amplifier, and a 16-bit and 1 MS/s data acquisition module interfaced to an RT cRIO controller. The cRIO controller not only executed DSP algorithms, signal digitalization, and the required PPM demodulation process but also transmitted the results and information to a personal computer via Ethernet to an IHM for displaying the attained results. A proprietary communication protocol at physical and data link layers, including real-time DSP filtering, PPM encoding, PPM decoding, and HASH byte validation was presented.

Experiments were carried out under laboratory conditions using 1 and 10 m 1K22 MP-35N multiwire cables. Results show that by exploring the waveguide effect of multi-conductor cables acting as a communication channel, successful transmission, reception, and validation of 60 kHz encoded PPM digital information has been achieved. The communication system and protocol show data transmission rates up to 470 bps with an average byte error rate around 5%. Although the trials have been conducted over small distances, the acoustic guided waves digital data transmission has been demonstrated by the feasibility of detecting and decoding in real-time dispersive guided waves energy packets, provided sufficient SNR. The implemented communication protocol suggests that a non-complex modulation, such as PPM, is sufficient to transmit digital information. Although data transmissions present occasional loss bits, increasing the robustness of the communication protocol recommends a better performance for the digital communication, for example, using bit codification techniques, redundancy, and adaptive modulations, which is proposed as future work. These results form the basis for future development of a guided waves data transmission system for automatic monitoring purposes using longer mono-conductor multiwire cables immersed in oil and water to resemble bottom hole conditions.
